# Proteins Binding to the Carbohydrate HNK-1: Common Origins?

**DOI:** 10.3390/ijms22158116

**Published:** 2021-07-29

**Authors:** Gaston Castillo, Ralf Kleene, Melitta Schachner, Gabriele Loers, Andrew E. Torda

**Affiliations:** 1 Center for Molecular Neurobiology, University Medical Center Hamburg-Eppendorf, Martinistr. 52, 20246 Hamburg, Germany; gaston.ecastillo@gmail.com (G.C.); ralf.kleene@zmnh.uni-hamburg.de (R.K.); 2 Keck Center for Collaborative Neuroscience, Department of Cell Biology and Neuroscience, Rutgers University, 604 Allison Road, Piscataway, NJ 08854, USA; schachner@dls.rutgers.edu; 3 Centre for Bioinformatics, University of Hamburg, Bundesstr. 43, 20146 Hamburg, Germany

**Keywords:** carbohydrate, development, HNK-1, receptor, sequence comparison

## Abstract

The human natural killer (HNK-1) carbohydrate plays important roles during nervous system development, regeneration after trauma and synaptic plasticity. Four proteins have been identified as receptors for HNK-1: the laminin adhesion molecule, high-mobility group box 1 and 2 (also called amphoterin) and cadherin 2 (also called N-cadherin). Because of HNK-1′s importance, we asked whether additional receptors for HNK-1 exist and whether the four identified proteins share any similarity in their primary structures. A set of 40,000 sequences homologous to the known HNK-1 receptors was selected and used for large-scale sequence alignments and motif searches. Although there are conserved regions and highly conserved sites within each of these protein families, there was no sequence similarity or conserved sequence motifs found to be shared by all families. Since HNK-1 receptors have not been compared regarding binding constants and since it is not known whether the sulfated or non-sulfated part of HKN-1 represents the structurally crucial ligand, the receptors are more heterogeneous in primary structure than anticipated, possibly involving different receptor or ligand regions. We thus conclude that the primary protein structure may not be the sole determinant for a bona fide HNK-1 receptor, rendering receptor structure more complex than originally assumed.

## 1. Introduction

The HNK-1 carbohydrate, also known as CD57 or LEU7, is a trisaccharide comprising glucuronic acid, galactose and N-acetyl-glucosamine. It can be terminally sulfated at the glucuronic acid (HSO_3_-3GlcAβ1-3Galβ1-4GlcNAc) [1] and is mainly expressed on glycolipids and glycoproteins in the nervous system in a controlled spatiotemporal pattern [2,3,4,5,6]. HNK-1 is amongst the glycans detected on several neural cell adhesion molecules, transmembrane receptors and extracellular matrix proteins in the nervous system [7,8,9,10,11,12] where it plays a role in diverse neural functions such as cell recognition, adhesion, migration, synaptic plasticity [5,6,11,13,14,15], preferential motor re-innervation and regeneration after injury of the peripheral and central nervous system [11,12,16,17,18,19,20,21]. HNK-1 has also been implicated in chronic infection and aging [22].

Despite the importance of the HNK-1 glycan in the nervous system, only a few proteins have been proposed as receptors. These are the laminins [15,23], high-mobility group box (HMGB) proteins 1 and 2 [24,25] (also called amphoterins [26]) and cadherin-2 (also known as neural cadherin) [12]. The different groups of proteins have been associated with different roles of HNK-1. Binding to laminins is involved in the adhesion of neurons and glial cells to the extracellular matrix as well as neural cell migration and outgrowth of processes from neurons and astrocytes [12,15,23,27,28,29]. Cadherin-2 is known for its role in cell adhesion, but more specifically, in binding to the α-amino-3-hydroxy-5-methylisoxazole propionate (AMPA)-type glutamate receptor subunit 2 (GluR2). Binding of cadherin-2 to GluR2 was shown to depend on the presence of HNK-1 on GluR2. GluR2 on the cell surface is stabilized by HNK-1, thus one can regard the HNK-1 glycan as a modulator of AMPA receptor trafficking and synaptic plasticity [12]. The high-mobility group box proteins are non-histone chromosomal proteins that bind HNK-1 on sulfated glycolipids and glycoproteins [30,31] and promote neurite outgrowth in an HNK-1 dependent manner [30].

Little is known about the mechanisms by which HNK-1 binds its receptors. Crystal structures are available for some regions of the HNK-1 receptors, but there are no structures of the receptors with the bound glycan and it is not even known whether the glycan binds in its sulfated or non-sulfated state. Hall et al. identified a 21-mer peptide in laminin, which inhibited binding of the full protein to HNK-1. This is consistent with competition between this peptide and the corresponding region of the G2 α-laminin domain for binding to HNK-1 [23,28].

Given this dearth of structural information about the binding domain/s, it was deemed interesting to see what can be found at the level of sequence analysis. Is there any evidence for a common ancestor protein or domain within the candidate HNK-1 receptors? A shared sequence motif could hint at a shared evolutionary history. This reasoning is based on the rationale that HNK-1 carrier proteins, receptors and enzymes generating related glycans are ancient proteins conserved in evolution. This is most clearly shown by some of the associated biochemical pathways. For example, the sulfotransferase responsible for catalysing the sulfation of glucuronic acid of HNK-1 has homologues in mammals, amphibians and bony fish [32,33,34]. Thus, the glycan, its synthesis pathway, or at least close evolutionary relatives have a long, conserved history.

If there are sequence signals (conservation of specific amino acids through evolution) connecting the putative HNK-1 receptor families, they are likely to be weak, so one needs an appropriate strategy to find them. Firstly, large families of sequences were gathered with a simple iterative psi-blast database search [35]. This approach runs the risk of “profile contamination” in which unrelated sequences become part of the set for the position-specific scoring matrix [36,37]. Rather than saving computer time, we allowed more than a dozen iterations, but only accepting homologues within extremely conservative expectation values. Given sets of related homologues, full-length protein sequences were downloaded and realigned using a progressive aligner.

Collecting and accurately realigning homologues is practical for some thousands of homologues, but not for the very large numbers encountered when combining sets of homologues together. Faced with these high numbers, the strategy was to reduce each subset while retaining the most even spread of representatives across sequence space. This means calculating a fast alignment for a family of proteins and saving the matrix of dissimilarities between pairs of sequences. The entries of this matrix were then sorted, and the list was visited, starting from the most similar pairs. From each pair of sequences, one member was removed. The net result is that one removes the redundancy from nearly identical sequences and for some number of representatives obtains the most informative set of representatives. This process is referred to as the reduction of a set of sequences in the methods, but the reduced sets still contained thousands of members as detailed under Methods.

For motif searches, we did not want to be limited to recognized motifs reported in the literature. Instead, we ran a full expectation-maximization search within each family and then between each family of candidate receptors [38]. Given the scale and sensitivity of the methods, one would expect that even remote similarities would be found.

## 2. Results

### 2.1. Alignment and Conservation of HNK-1 Receptors

The aim was not to study the individual putative HNK-1 receptors, but to find features common to the different receptor families. The first important result came from an attempt to align all three families (15,000 sequences, 5000 per family). One cannot show the whole alignment, since it is essentially a mass of unaligned, gapped sequences. A neighbor-joining tree was calculated and showed three unrelated groups of proteins. There is no similarity between the families, with the exception of HMGB-1 and HMBG-2. These can be aligned and from here are treated as just one group. Although not shown, the alignments were tried with local and global alignments, as well as various subsets. This changes how we regard the receptors. There are three distinct families. Although one can align the members of each family, there is no plausible alignment spanning the postulated HNK-1 receptor groups.

**Figure 1:**
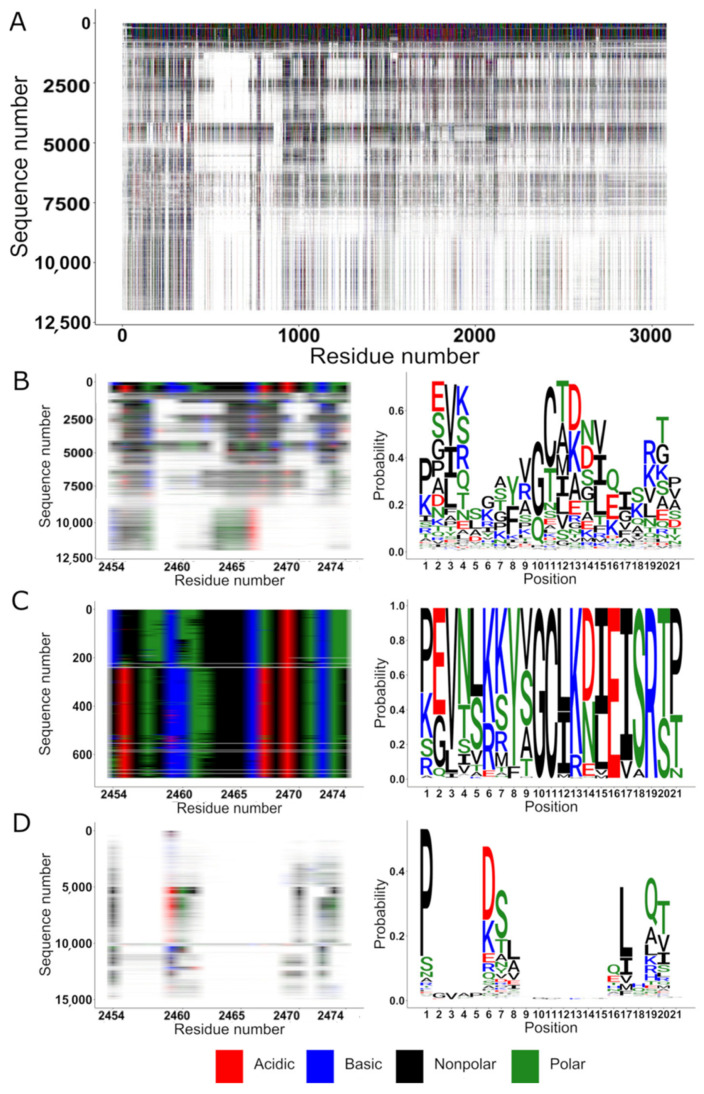
HNK-1 binding site conservation within the laminin-1 family members. (**A**) Sequence alignment of 11,999 similar sequences to laminin-1. From the multiple sequence alignment, columns are only shown for sites present in laminin-1. Acidic residues are represented in red, basic in blue, nonpolar in black, polar in green and gaps in white. (**B**) Conservation within the proposed HNK-1 binding site of laminin-1 (residues 2454–2474), multiple alignment overview (**left**) and sequence logo (**right**). The size of the letters is proportional to the fraction of the residue (***y*-axis**) found in a certain position of the alignment (***x*-axis**). (**C**) The same region of, but focusing on the 700 most similar sequences, multiple alignment overview (**left**) and sequence logo (**right**). (**D**) Laminin-1 residues 2454–2474 conservation inside the 15,000-sequence alignment of the three HNK-1 binding proteins.

Conserved sites and regions can be seen within each group and compared to the literature. The overall conservation of laminin-α is shown in Figure 1. In the upper diagram, columns were removed if no residue was present in laminin-α. One can see that the closest several hundred homologues are readily alignable, but the more remote homologues are missing entire domains and there is a first domain that is always present. We then focused on residues 2454–2474 within which an HNK-1 binding site has been reported [23]. Looking at the whole sequence set, there are large gaps (white). This suggests that if this is an HNK-1 binding site, it only serves this role in mammals or it only involves the sites which are generally present in the molecule. One can further concentrate on the 700 closest sequences. They are mostly from mammals (50%), birds (22%) and ray-finned fish (20%) with the remainder from reptilians, amphibians and others. To return to the original search for similarities amongst the different sequence families, one should note that there is no similarity or meaningful alignment with residues outside of the laminin group. The lack of overall similarity between the groups of proteins does not preclude the existence of smaller shared sequence motifs.

### 2.2. Motifs Common to All Families

Given the cost of full motif searches, we began by reducing the sequence set to 3 × 400 = 1200 from the original 3 × 5000. A truly universal motif would appear in the full set of 1200 sequences, but a motif could be shared by two of the families, so we decided to run motif searches over the (3 × 2)/2 = 3 pairs of families. Motif searches within each family were calculated for completeness and comparison with the literature.

For the full set of 1200 sequences, no statistically significant motifs were found over the complete set. For each pair of families (800 sequences), no motifs were found spanning both families of the pair. There are, however, motifs that are unique to each family and that are also found in the literature as in the case of the cadherin CA domains [39], the high-mobility group (HMG) box domains [40] or the laminin-1 G domains [41].

Since a 21-mer segment in laminin-α (residues 2454–2474) binding HNK-1 was described [15,23], it was studied in more detail. Within the laminins, there is a highly significant (*e* value = 6.4 × 10^−323^) 21 amino acid motif (residues 2457–2477) which overlaps, but falls outside of the region by three residues. This exact motif occurs five times within the laminins and corresponds to the C-terminus of the laminin G domains, extending for the five repetitions by four residues. The HNK-1 interacting sequence in the second laminin G domain [23] is not shared with the other putative HNK-1 receptors.

**Figure 2:**
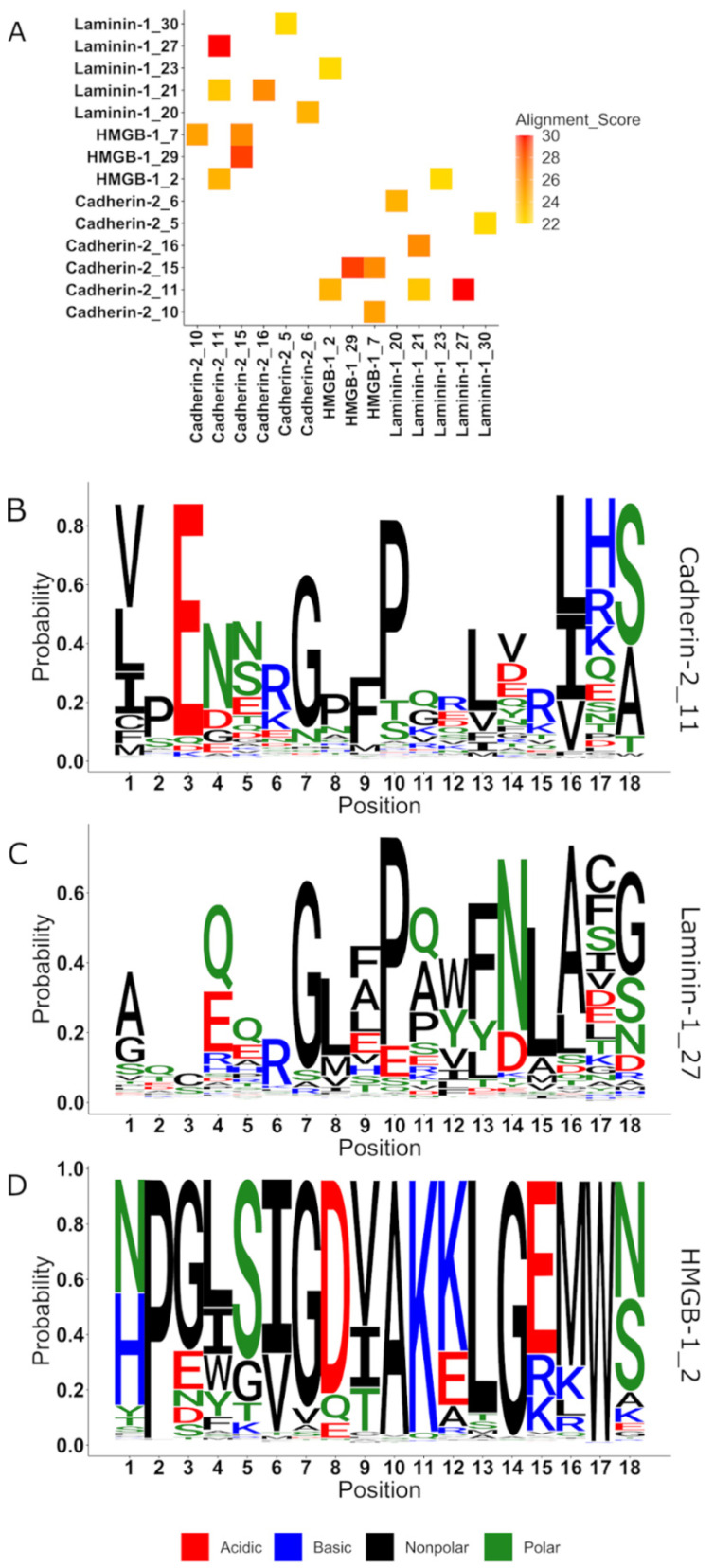
Sliding window comparison between the 90 motifs found in the three families of HNK-1 receptors. (**A**) Heat map depicting the 10 most similar motifs alignments between two motifs from different HNK-1 binding families. The higher the alignment score, the more similar are the sequences. (**B**) Sequence logo of the aligned similar sequences to cadherin-2 corresponding to the residues 168 to 185 and the 11th cadherin-2 family motif (arranged from 1 as the most probable to 30 less probable). (**C**) Sequence logo of the aligned similar sequences to laminin-1 corresponding to the residues 22 to 39 and the 27th laminin-1 family motif. (**D**) Sequence logo of the aligned similar sequences to HMGB-1 corresponding to the residues 117 to 134 and the second HMGB-1 family motif.

We found three motifs with similar *e*-values < 10^−40^ in the different HNK-1 receptor families corresponding to cadherin-2 motif 11, laminin-1 motif 27 and HMGB-1 motif 2 (Figure 2). The three families share a common glycine in position 7 of the alignment depicted in the sequence logos (Figure 2) followed by non-polar residues at position 9, 10, 13 and 16. Yet, this weak similarity was not found to be biologically meaningful.

## 3. Discussion

A motif shared by the different putative HNK-1 binding proteins would be consistent with the belief that several different protein families directly bind HNK-1. This would be a signal that one could look for in other proteins, which might have roles in neural functions. There are certain sites conserved within each family. There are even motifs repeated within some families. We, however, found absolutely no evidence for a motif common to the different families. The sequence searches, alignments and motif searches used here were rather large and should have been able to detect any plausible statistical signal.

Based on the finding on a specific HNK-1 binding site within a 21-mer stretch of mouse laminin-1 [23], we looked for similar stretches in other proteins. From the closest 700 homologues, one can extract the corresponding consensus sequence (RXX[VL]XX[KR][KR]YXGC[LI][KR]X[LI]EISR[TS]). This sequence contains four basic amino acids, which might be involved in binding sulfated HNK-1, since basic residues are involved in binding of matriglycan, a polysaccharide on dystroglycan, to the fourth laminin G domain [42,43]. Continuing in this vein, one should not ignore the arguments made for the importance of interactions of aliphatic protons with π-electron systems, specifically in protein-oligo/polysaccharide systems [44,45,46]. In a protein as long as laminin, there are many 21-mer fragments with several basic residues or even one isolated individual tyrosine. We therefore proposed that it may be more useful to make a simple and testable prediction. There is a repeated motif, which overlaps the 21-mer sequence region and occurs five times (in each of the laminin G domains), but only the second laminin G domain was found to bind HNK-1. There are two possible explanations. Either the quaternary domain arrangement restricts accessibility in four copies, or the binding is sensitive to small changes in sequence. Both explanations are plausible, considering observations of different binding partners for different laminin G domains [47,48]. A previous study modelling the second laminin G domain reported no deep binding pocket that is characteristic of HNK-1 binding [49], which is in agreement with our results.

Looking for hidden similarities, we found top 10 motifs from each of the three possible receptor families when compared with a sliding window (Figure 2), but even in this case, no similarity could be seen within the self-imposed limitation to common motifs in primary structure.

Flexibility of oligomeric/polymeric glycans with their structural heterogeneity is problematic for the search of receptors. It is highly possible that different receptors bind to different conformations or different chemical states. From a biochemical point of view, it is not known whether HNK-1 acts in the sulfated or non-sulfated form. At a physiological pH, these forms are subject to considerable structural differences, particularly since the effects of sulfation are difficult to predict from antibody studies: some antibodies bind to one form of the glycan and some to both forms [9,50,51]. Given these considerations, one has to assume that one glycan may be bound by different environments in different proteins. Taking mannose/protein binding as an example, different constraints have been proposed for human immunodeficiency virus (HIV) [52,53], the mannose-binding lectin CD4 as well as dendritic cell-specific intercellular adhesion molecule 3-grabbing non-integrin (DC-SIGN) [54,55].

Although evidence of HNK-1 binding capacities has been based on antibody studies, there is no doubt that HNK-1 binds directly, for instance, to laminin, since the binding partners were purified molecules. However, some proteins considered to be HNK-1 binders may bind via a third molecule, which is directly bound to HNK-1. Precedents for this argument could be that glycan-binding associated with novoviruses involves more than a single protein and well-defined glycan [56]. From our results, we have to conclude that each receptor protein family has its own way of binding to the HNK-1 glycan and although the three broad families studied have strongly conserved regions, no striking sequence features were common to all families.

Since co-crystallography of proteins with glycans is a very difficult undertaking because of the high flexibility of glycans, one may invest in NMR studies, as exemplified by studies on the interaction of a lipopolysaccharide from *Klebsiella pneumoniae* with lysozyme [57]. Unfortunately, a single laminin domain is five times larger than lysozyme. Thus, chemical shift titration and saturation-transfer techniques can only yield overall binding in the same way as surface plasmon resonance or scanning microcalorimetry [58,59]. For the moment, the details of HNK-1 binding remain an area ripe for speculation. We expect that a realistic goal would be to document the different binding behavior of the sulfated and non-sulfated forms of the HNK-1 glycan using the identified HNK-1 binding domain of laminin as a first step to gain deeper insights into the remarkable functional versatility of HNK-1.

## 4. Materials and Methods

Sequences for each of the putative HNK-1 binding proteins are given in Table 1. Homologues were collected using psi-blast [35] with up to 30 iterations and accepting homologues with an expectation value of less than 10^−21^, which was near the most conservative value possible, while allowing the calculations to finish in practical time.

MAFFT [60] was used for all multiple sequence alignments. Initial sequence alignments for laminins and cadherin were run in default (fast) mode with a maximum of five iterations. For alignments for the HMGB family (HMGB1 and HMG2) with fewer homologues, MAFFT was run in its most accurate mode with affine gap penalties.

After initial data collection and alignment, each of the HNK-1 receptor families was reduced to 5000 representative sequences [61]. These were combined to form a full set of 15,000 sequences covering all protein families and a full alignment was calculated—again in fast mode.

From each protein family, several thousand (Table 1) homologues were used for conservation calculations after a second cut-off was applied. Sequences were re-aligned, and variability was calculated using Shannon entropy *S_i_* at each site i

(1) $$S_{i}=\overset{a=20}{\underset{a=1}{\sum }}p_{a}\log_{20}p_{a}$$

where *p_α_* is the probability of amino acid type a in a column of the alignment and the summation runs over the 20 amino acid types [62,63].

Literature domains were taken from SMART (Simple Modular Architecture Research Tool) [64,65]. MEME was used to search for sequence motifs using expectation maximization and to estimate probabilities of chance occurrence [38,66]. Because of the cost of the calculations, each aligned family of sequences was reduced to 400 representatives. The minimum and maximum lengths for motifs were set to 6 and 30 residues. The pairwise alignment function from the Biostrings R package [67] was used to display the relation between two sequences.

Numbers are given as e.g., 10,000 as in the ACS Chicago layout regulations despite the guides for nomenclature of numbers in scientific documents [68,69].

**Figure 3:**
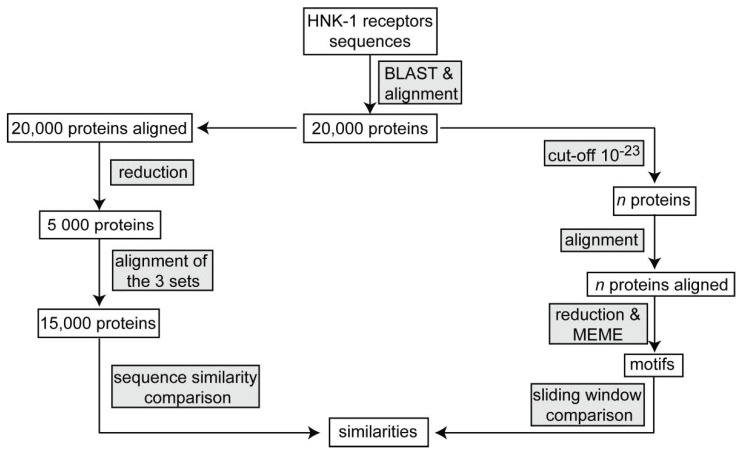
Flowchart of the steps involved in the study of the HNK-1 receptors. The workflow comprises two approaches using the results of the alignment of 20,000 proteins of each family (**left branch**). The 20,000 sequences were reduced to 5000 representative sequences and merged to form a 15,000 sequences group to be aligned. In the second approach (**right branch**)—after a second cut-off was applied (see also Table 1)—families were realigned and then used to find conserved sites.

The steps are summarized in Figure 3.

## Figures and Tables

**Table 1:** Proteins used for initial sequence searches. ID is the gene identifier. Sequence length gives the number of amino acids in the query sequence. *n* homologues are the number of homologues accepted for the motif identification.

	ID	Sequence Length	*n* Homologues
Cadherin-2	NP_031690.3	906	14,128
HMGB-1	NP_034569.1	215	1590
HMGB-2	NP_032278.1	199	1573
Laminin (subunit α-1)	NP_032506.2	3083	11,999

## Data Availability

All data were taken from public databanks.

## Abbreviations

AMPA	α-amino-3-hydroxy-5-methyl-4-isoxazolepropionic acid
GluR2	glutamate receptor 2
HMGB	high-mobility group box
HNK	human natural killer

## References

[1] Sheikh M., Venzke D., Anderson M.E., Yoshida-Moriguchi T., Glushka J.N., Nairn A.V., Galizzi M., Moremen K.W., Campbell K.P., Wells L. (2020). HNK-1 sulfotransferase modulates α-dystroglycan glycosylation by 3-O-sulfation of glucuronic acid on matriglycan. Glycobiology.

[2] Schwarting G.A., Jungalwala F.B., Chou D.K., Boyer A.M., Yamamoto M. (1987). Sulfated glucuronic acid-containing glycoconjugates are temporally and spatially regulated antigens in the developing mammalian nervous system. Dev. Biol..

[3] Chou D.K.H., Schwarting G.A., Evans J.E., Jungalwala F.B. (1987). Sulfoglucuronyl-neolacto series of glycolipids in peripheral nerves reacting with HNK-1 antibody. J. Neurochem..

[4] Chou D.K., Ilyas A.A., Evans J.E., Costello C., Quarles R.H., Jungalwala F.B. (1986). Structure of sulfated glucuronyl glycolipids in the nervous system reacting with HNK-1 antibody and some IgM paraproteins in neuropathy. J. Biol. Chem..

[5] Sytnyk V., Leshchyns’Ka I., Schachner M. (2020). Neural glycomics: The sweet side of nervous system functions. Cell. Mol. Life Sci..

[6] Kizuka Y., Oka S. (2012). Regulated expression and neural functions of human natural killer-1 (HNK-1) carbohydrate. Cell. Mol. Life Sci..

[7] Poltorak M., Sadoul R., Keilhauer G., Landa C., Fahrig T., Schachner M. (1987). Myelin-associated glycoprotein, a member of the L2/HNK-1 family of neural cell adhesion molecules, is involved in neuron-oligodendrocyte and oligodendrocyte-oligodendrocyte interaction. J. Cell Biol..

[8] Martini R., Schachner M. (1986). Immunoelectron microscopic localization of neural cell adhesion molecules (L1, N-CAM, and MAG) and their shared carbohydrate epitope and myelin basic protein in developing sciatic nerve. J. Cell Biol..

[9] Kruse J., Mailhammer R., Wernecke H., Faissner A., Sommer I., Goridis C., Schachner M., Kruse J., Mailhammer R., Wernecke H. (1984). Neural cell adhesion molecules and myelin-associated glycoprotein share a common carbohydrate moiety recognized by monoclonal antibodies L2 and HNK-1. Nat. Cell Biol..

[10] Kruse J., Keilhauer G., Faissner A., Timpl R., Schachner M. (1985). The J1 glycoprotein—A novel nervous system cell adhesion molecule of the L2/HNK-1 family. Nature.

[11] Morise J., Takematsu H., Oka S. (2017). The role of human natural killer-1 (HNK-1) carbohydrate in neuronal plasticity and disease. Biochim. Biophys. Acta (BBA)-Gen. Subj..

[12] Morita I., Kakuda S., Takeuchi Y., Itoh S., Kawasaki N., Kizuka Y., Kawasaki T., Oka S. (2009). HNK-1 Glyco-epitope regulates the stability of the glutamate receptor subunit GluR2 on the neuronal cell surface. J. Biol. Chem..

[13] Schachner M., Martini R., Hall H., Orberger G. (1995). Functions of the L2/HNK-1 carbohydrate in the nervous system. Prog. Brain. Res..

[14] Jungalwala F.B. (1994). Expression and biological functions of sulfoglucuronyl glycolipids (SGGLs) in the nervous system? A review. Neurochem. Res..

[15] Hall H., Liu L., Schachner M., Schmitz B. (1993). The L2/HNK-1 carbohydrate mediates adhesion of neural cells to laminin. Eur. J. Neurosci..

[16] Yabuno K., Morise J., Kizuka Y., Hashii N., Kawasaki N., Takahashi S., Miyata S., Izumikawa T., Kitagawa H., Takematsu H. (2015). A sulfated glycosaminoglycan linkage region is a novel type of human natural killer-1 (HNK-1) epitope expressed on aggrecan in perineuronal nets. PLoS ONE.

[17] Saghatelyan A., Gorissen S., Albert M., Hertlein B., Schachner M., Dityatev A. (2000). The extracellular matrix molecule tenascin-R and its HNK-1 carbohydrate modulate perisomatic inhibition and long-term potentiation in the CA1 region of the hippocampus. Eur. J. Neurosci..

[18] Martini R., Schachner M., Brushart T.M. (1994). The L2/HNK-1 carbohydrate is preferentially expressed by previously motor axon-associated Schwann cells in reinnervated peripheral nerves. J. Neurosci..

[19] Low K., Orberger G., Schmitz B., Martini R., Schachner M. (1994). The L2/HNK-1 carbohydrate is carried by the myelin associated glycoprotein and sulphated glucuronyl glycolipids in muscle but not cutaneous nerves of adult mice. Eur. J. Neurosci..

[20] Simova O., Irintchev A., Mehanna A., Liu J., Dihné M., Bächle D., Sewald N., Loers G., Schachner M. (2006). Carbohydrate mimics promote functional recovery after peripheral nerve repair. Ann. Neurol..

[21] Sahu S., Li R., Kadeyala P.K., Liu S., Schachner M. (2018). The human natural killer-1 (HNK-1) glycan mimetic ursolic acid promotes functional recovery after spinal cord injury in mouse. J. Nutr. Biochem..

[22] Kared H., Martelli S., Ng T.P., Pender S.L., Larbi A. (2016). CD57 in human natural killer cells and T-lymphocytes. Cancer Immunol. Immunother..

[23] Hall H., Vorherr T., Schachner M. (1995). Characterization of a 21 amino acid peptide sequence of the laminin G2 domain that is involved in HNK-1 carbohydrate binding and cell adhesion. Glycobiology.

[24] Chou D.K.H., Zhang J., Smith F.I., McCaffery P., Jungalwala F.B. (2004). Developmental expression of receptor for advanced glycation end products (RAGE), amphoterin and sulfoglucuronyl (HNK-1) carbohydrate in mouse cerebellum and their role in neurite outgrowth and cell migration. J. Neurochem..

[25] Fang P., Schachner M., Shen Y.-Q. (2012). HMGB1 in development and diseases of the central nervous system. Mol. Neurobiol..

[26] Rauvala H., Rouhiainen A. (2010). Physiological and pathophysiological outcomes of the interactions of HMGB1 with cell surface receptors. Biochim. Biophys. Acta Gene Regul. Mech..

[27] Schmidt J.T., Schachner M. (1998). Role for cell adhesion and glycosyl (HNK-1 and oligomannoside) recognition in the sharpening of the regenerating retinotectal projection in goldfish. J. Neurobiol..

[28] Hall H., Deutzmann R., Timpl R., Vaughan L., Schmitz B., Schachner M. (1997). HNK-1 carbohydrate-mediated cell adhesion to laminin-1 is different from heparin-mediated and sulfatide-mediated cell adhesion. JBIC J. Biol. Inorg. Chem..

[29] Schmitz B., Schachner M., Ito Y., Nakano T., Ogawa T. (1994). Determination of structural elements of the L2/HNK-1 carbohydrate epitope required for its function. Glycoconj. J..

[30] Chou D.K., Evans J.E., Jungalwala F.B. (2001). Identity of nuclear high-mobility-group protein, HMG-1, and sulfoglucuronyl carbohydrate-binding protein, SBP-1, in brain. J. Neurochem..

[31] Chou D.K.H., Henion T.R., Jungalwala F.B. (2003). Regulation of expression of sulfoglucuronyl carbohydrate (HNK-1), Amphoterin and RAGE in retinoic acid-differentiated P19 embryonal carcinoma cells. J. Neurochem..

[32] Morita I., Kizuka Y., Kakuda S., Oka S. (2007). Expression and function of the HNK-1 carbohydrate. J. Biochem..

[33] Ma L., Shen H.-F., Shen Y.-Q., Schachner M. (2016). The adhesion molecule-characteristic HNK-1 carbohydrate contributes to functional recovery after spinal cord injury in adult zebrafish. Mol. Neurobiol..

[34] Lang D.M., Stuermer C.A. (1996). Adaptive plasticity of *Xenopus glial* cells in vitro and after CNS fiber tract lesions in vivo. Glia.

[35] Altschul S.F., Madden T.L., Schäffer A.A., Zhang J., Zhang Z., Miller W., Lipman D.J. (1997). Gapped BLAST and PSI-BLAST: A new generation of protein database search programs. Nucleic Acids Res..

[36] Li W., McWilliam H., Goujon M., Cowley A., Lopez R., Pearson W.R. (2012). PSI-Search: Iterative HOE-reduced profile SSEARCH searching. Bioinformatics.

[37] Pearson W., Li W., López R. (2016). Query-seeded iterative sequence similarity searching improves selectivity 5–20-fold. Nucleic Acids Res..

[38] Bailey T.L., Elkan C. (1995). Unsupervised learning of multiple motifs in biopolymers using expectation maximization. Mach. Learn..

[39] Shapiro L., Kwong P.D., Fannon A.M., Colman D.R., Hendrickson W.A. (1995). Considerations on the folding topology and evolutionary origin of cadherin domains. Proc. Natl. Acad. Sci. USA.

[40] Reeves R., Nissen M.S. (1990). The A.T-DNA-binding domain of mammalian high mobility group I chromosomal proteins. A novel peptide motif for recognizing DNA structure. J. Biol. Chem..

[41] Beckmann G., Hanke J., Bork P., Reich J.G. (1998). Merging extracellular domains: Fold prediction for laminin G-like and amino-terminal thrombospondin-like modules based on homology to pentraxins. J. Mol. Biol..

[42] Hohenester E. (2019). Structural biology of laminins. Essays Biochem..

[43] Briggs D., Yoshida-Moriguchi T., Zheng T., Venzke D., Anderson M.E., Strazzulli A., Moracci M., Yu L., Hohenester E., Campbell K.P. (2016). Structural basis of laminin binding to the LARGE glycans on dystroglycan. Nat. Chem. Biol..

[44] Zhang R., Loers G., Schachner M., Boelens R., Wienk H., Siebert S., Eckert T., Kraan S., Rojas-Macias M.A., Lütteke T. (2016). Molecular basis of the receptor interactions of polysialic acid (polySia), polySia mimetics, and sulfated polysaccharides. ChemMedChem.

[45] Spiwok V. (2017). CH/π Interactions in carbohydrate recognition. Molecules.

[46] Hudson K.L., Bartlett G.J., Diehl R.C., Agirre J., Gallagher T., Kiessling L.L., Woolfson D.N. (2015). Carbohydrate–aromatic interactions in proteins. J. Am. Chem. Soc..

[47] Tisi D., Talts J.F., Timpl R., Hohenester E. (2000). Structure of the C-terminal laminin G-like domain pair of the laminin alpha 2 chain harbouring binding sites for alpha -dystroglycan and heparin. EMBO J..

[48] Ichikawa N., Kasai S., Suzuki N., Nishi N., Oishi S., Fujii N., Kadoya Y., Hatori K., Mizuno Y., Nomizu M. (2005). Identification of neurite outgrowth active sites on the laminin alpha4 chain G domain. Biochemistry.

[49] Bhunia A., Vivekanandan S., Eckert T., Burg-Roderfeld M., Wechselberger R., Romanuka J., Bächle D., Kornilov A.V., von der Lieth C.W., Jiménez-Barbero J. (2010). Why structurally different cyclic peptides can be glycomimetics of the HNK-1 carbohydrate antigen. J. Am. Chem. Soc..

[50] Abo T., Balch C.M. (1981). A differentiation antigen of human NK and K cells identified by a monoclonal antibody (HNK-1). J. Immunol..

[51] Noronha A.B., Ilyas A., Antonicek H., Schachner M., Quarles R.H. (1986). Molecular specificity of L2 monoclonal antibodies that bind to carbohydrate determinants of neural cell adhesion molecules and their resemblance to other monoclonal antibodies recognizing the myelin-associated glycoprotein. Brain Res..

[52] Tamura M., Natori K., Kobayashi M., Miyamura T., Takeda N. (2004). Genogroup II noroviruses efficiently bind to heparan sulfate proteoglycan associated with the cellular membrane. J. Virol..

[53] Taube S., Perry J.W., Yetming K., Patel S.P., Auble H., Shu L., Nawar H.F., Lee C.H., Connell T.D., Shayman J.A. (2009). Ganglioside-linked terminal sialic acid moieties on murine macrophages function as attachment receptors for murine noroviruses. J. Virol..

[54] Ji X., Gewurz H., Spear G.T. (2005). Mannose binding lectin (MBL) and HIV. Mol. Immunol..

[55] Marzi A., Mitchell D.A., Chaipan C., Fisch T., Doms R.W., Carrington M., Desrosiers R.C., Pöhlmann S. (2007). Modulation of HIV and SIV neutralization sensitivity by DC-SIGN and mannose-binding lectin. Virology.

[56] Neu U., Bauer J., Stehle T. (2011). Viruses and sialic acids: Rules of engagement. Curr. Opin. Struct. Biol..

[57] Zhang R., Wu L., Eckert T., Burg-Roderfeld M., Rojas-Macias M.A., Lütteke T., Krylov V.B., Argunov D.A., Datta A., Markart P. (2017). Lysozyme’s lectin-like characteristics facilitates its immune defense function. Q. Rev. Biophys..

[58] Battistel M.D., Azurmendi H.F., Yu B., Freedberg D.I. (2014). NMR of glycans: Shedding new light on old problems. Prog. Nucl. Magn. Reson. Spectrosc..

[59] Tsvetkov Y.E., Burg-Roderfeld M., Loers G., Arda A., Sukhova E.V., Khatuntseva E.A., Grachev A.A., Chizhov A.O., Siebert H.-C., Schachner M. (2011). Synthesis and molecular recognition studies of the HNK-1 trisaccharide and related oligosaccharides. The specificity of monoclonal anti-HNK-1 antibodies as assessed by surface plasmon resonance and STD NMR. J. Am. Chem. Soc..

[60] Katoh K., Standley D.M. (2013). MAFFT multiple sequence alignment software version 7: Improvements in performance and usability. Mol. Biol. Evol..

[61] Torda A.E. (2020). Reduce ACM. Sigsam Bull..

[62] Strait B., Dewey T., Strait B., Dewey T. (1996). The Shannon information entropy of protein sequences. Biophys. J..

[63] Torda A.E. (2019). Entropy.pl. https://zenodo.org/record/4334283#.YQICgI4zZPY.

[64] Letunic I., Doerks T., Bork P. (2015). SMART: Recent updates, new developments and status in 2015. Nucleic Acids Res..

[65] Letunic I., Bork P. (2017). 20 years of the SMART protein domain annotation resource. Nucleic Acids Res..

[66] Bailey T.L., Boden M., Buske F.A., Frith M., Grant C.E., Clementi L., Ren J., Li W.W., Noble W.S. (2009). MEME SUITE: Tools for motif discovery and searching. Nucleic Acids Res..

[67] Pagès H.A.P., Gentleman R., DebRoy S. (2019). Biostrings: Efficient manipulation of biological strings. R Package Version.

[68] https://nam02.safelinks.protection.outlook.com/?url=https%3A%2F%2Fwww.nist.gov%2Fpml%2Fspecial-publication-811%2Fnist-guide-si-chapter-10-more-printing-and-using-symbols-and-numbers%231053&data=04%7C01%7Cschachner%40dls.rutgers.edu%7Cf4bf2bb292e443e2fd0008d95029d375%7Cb92d2b234d35447093ff69aca6632ffe%7C1%7C1%7C637628962842219163%7CUnknown%7CTWFpbGZsb3d8eyJWIjoiMC4wLjAwMDAiLCJQIjoiV2luMzIiLCJBTiI6Ik1haWwiLCJXVCI6Mn0%3D%7C3000&sdata=Dnky8qDcpBI2rPm0OhZChlDaBWbETQN740X2Uun%2BdXg%3D&reserved=0.

[69] https://nam02.safelinks.protection.outlook.com/?url=https%3A%2F%2Fwww.bipm.org%2Fen%2Fcommittees%2Fcg%2Fcgpm%2F22-2003%2Fresolution-10&data=04%7C01%7Cschachner%40dls.rutgers.edu%7Cf4bf2bb292e443e2fd0008d95029d375%7Cb92d2b234d35447093ff69aca6632ffe%7C1%7C1%7C637628962842219163%7CUnknown%7CTWFpbGZsb3d8eyJWIjoiMC4wLjAwMDAiLCJQIjoiV2luMzIiLCJBTiI6Ik1haWwiLCJXVCI6Mn0%3D%7C3000&sdata=LWQDiDaVZyeynw9lqgDClv%2FVCpoWgjkJL7Vkye0x9Bg%3D&reserved=0.

